# Concerns about the consequences of cancer predisposition and relationships with quality of life in young adults with Li-Fraumeni syndrome

**DOI:** 10.1038/s41431-025-01984-w

**Published:** 2025-11-23

**Authors:** Rowan Forbes Shepherd, Camella J. Rising, Richard P. Moser, Chloe O. Huelsnitz, Patrick Boyd, Catherine Wilsnack, Alix G. Sleight, William M. P. Klein, Allison Werner-Lin, Sadie P. Hutson, Paul K. J. Han, Payal P. Khincha

**Affiliations:** 1 Clinical Genetics Branch, Division of Cancer Epidemiology and Genetics, National Cancer Institute, Rockville, MD USA; 2 Behavioral Research Program, Office of the Associate Director, Division of Cancer Control and Population Sciences, National Cancer Institute, Rockville, MD USA; 3 Department of Population Sciences, Clinical Cancer Genomics, City of Hope, Duarte, CA USA; 4 Steve Hicks School of Social Work, University of Texas at Austin, Austin, TX USA; 5 Division of Occupational Science and Occupational Therapy, University of Southern California, Los Angeles, CA USA; 6 School of Social Policy and Practice, University of Pennsylvania, Philadelphia, PA USA; 7 Division of Nursing, The University of Texas MD Anderson Cancer Center, Houston, TX USA

**Keywords:** Risk factors, Psychology, Cancer genetics

## Abstract

Li-Fraumeni syndrome (LFS) is an inherited condition associated with high multi-organ cancer risks from birth. Young adults (YAs; 18–39 years) with LFS experience psychosocial challenges that may negatively affect health-related quality of life (HRQOL). This study aimed to describe a specific psychosocial challenge, concerns about the consequences of LFS (CC-LFS), and investigate relationships among CC-LFS and HRQOL among YAs. An online survey assessed CC-LFS and HRQOL among YAs with LFS enrolled in a National Cancer Institute study. Regression analyses examined relationships between CC-LFS and HRQOL scores, adjusted for age, cancer, and history of mental health challenges. Of 37 total respondents (78% female; *M*_age_ = 31 years), 51% had a cancer history. Many reported past mental health challenges, including emotional problems (70%), depression/anxiety disorders (68%), and suicidal ideation (43%). Mean scores for mental (*M* = 65.8), physical (*M* = 67.5), and general (*M* = 63.9) HRQOL were lower than in the general population. Higher CC-LFS scores (*M* = 31/50, SD = 7.2) were associated with lower scores for each HRQOL domain. Adjusting for covariates, higher CC-LFS scores were associated with lower physical HRQOL (β = −0.491, 95% CI −0.76, −0.22, *p* < 0.001) and general HRQOL (β = −0.466, 95% CI −0.77, −0.16, *p* = 0.004), but not mental HRQOL (*p* = 0.102). CC-LFS accounted for significant proportions of unique variance in general (*R*^2^_adj_ = 17.7%, *p* = 0.004) and physical HRQOL (*R*^2^_adj_ = 20.4%, *p* < 0.001). Findings suggest YAs with LFS may experience substantial concerns, mental health challenges, and diminished HRQOL. Concerns about LFS consequences appear to be a psychosocial risk factor that clinicians and researchers might address through targeted interventions to improve YAs’ psychosocial health.

## Introduction

LFS is an autosomal dominant, early-onset cancer predisposition syndrome caused by pathogenic/likely pathogenic germline variants in *TP53*. Individuals with LFS have 24 times increased risk of any cancer compared to the general population, with the highest risk period from birth to 30 years [1]. Frequently occurring cancers in LFS include early-onset breast cancers, sarcomas, brain tumors, and adrenal cancers, among many others [2]. LFS also confers an elevated risk of developing multiple primary cancers over the life course [2]. Current clinical recommendations for LFS include intensive, multimodal whole-body cancer screening to detect cancers early [3, 4].

Young people with LFS, such as adolescents and young adults (AYAs, aged 15- 39 years), report experiencing many psychosocial challenges, including cancer worry, distress, anxiety, depression [5, 6], and diminished health-related quality of life (HRQOL) [7]. They also report various concerns regarding the consequences of living with high lifetime genetic cancer risk. These include, for example, concerns about genetic testing [8] and reproductive decision-making [9], LFS communication in family relationships [10], and future educational attainment or employment given the uncertainty of LFS-related cancer diagnoses [11]. Additional concerns include lifelong, whole-body cancer risks [8, 12–15], repeated cancer screening [13, 16, 17], and the psychological burden of potentially multiple personal or family member cancer diagnoses [18, 19].

Research focused on individuals with non-LFS cancer syndromes [20–22] suggests that concerns about the consequences of cancer predisposition may negatively affect the mental health and HRQOL of young people with LFS. Nested within the AYA age range, young adults (YAs; aged 18–39 years) may be especially vulnerable to LFS-related psychosocial sequelae given the compounding pressures of age-normative developmental experiences (e.g., self-discovery, dating, job or residential transitions). Although the psychosocial consequences of living with LFS have been explored in qualitative-descriptive studies [8–11, 13, 23–26], many have not focused on YAs, measured the extent of concerns about the consequences of LFS, or examined relationships among these concerns, mental health, and HRQOL.

A current challenge in addressing this research gap is the lack of measures to quantify enduring concerns about the consequences of cancer predisposition. Measures such as the Multidimensional Impact of Cancer Risk Assessment (MICRA) [27] and the Psychosocial Aspects of Hereditary Cancer questionnaire (PAHC) [28] assess several hereditary cancer concerns; however, both measures include items that are highly specific to genetic testing and counseling. The Genetic Psychosocial Risk Instrument (GPRI) is a 20-item screening tool designed for use in clinical genetics settings that assesses psychosocial risk factors among individuals undergoing genetic testing for adult-onset hereditary disease [29]. The GPRI is a promising measure to evaluate hereditary cancer concerns in the YA LFS population as items are applicable, or easily adaptable, for assessment of concerns that may continue or manifest long after genetic testing. Moreover, although not yet tested in the YA LFS population, the GPRI has been tested in multiple populations with hereditary cancer predisposition syndromes (e.g., Lynch, hereditary breast and ovarian cancer) and exhibited good psychometric properties in several studies [29–31].

This study had two primary aims. The first aim was to examine **c**oncerns about the **c**onsequences of **LFS** (CC-LFS) in the YA LFS population, utilizing the GPRI adapted for the LFS context. Our second aim was to investigate relationships between CC-LFS and HRQOL, controlling for history of mental health challenges and other clinical-demographic factors (e.g., age, prior cancer history). We hypothesized that, controlling for other factors, CC-LFS would be negatively associated with HRQOL.

## Subjects and methods

### Participants and procedure

Individuals enrolled in the National Cancer Institute’s (NCI) IRB-approved LFS Study (NCT01443468) were invited by email to complete a Qualtrics online survey, which was administered between February and September 2021. Participants were eligible for the survey study if they had a confirmed germline pathogenic or likely pathogenic *TP53* variant on clinical genetic testing and were aged 18–39 years. Participants provided consent via an online form before beginning the survey, which took less than 30 minutes to complete.

### Measures

#### Psychosocial variables

Complete versions of all psychosocial measures are included in Supplementary Material S3.

##### Concerns about the consequences of LFS

We measured CC-LFS by adapting the GPRI [29], selecting ten items that focused on concerns about personal and familial consequences of cancer predisposition (e.g., impact of future disease, implications of genetic risk), based on existing evidence of the salience of such concerns among young people living with LFS [8–11]. We slightly modified items from the original, for example, changing “the disease” to “cancer,” “cancer risk,” or “LFS,” as appropriate. Items included, for example, “I am concerned about my risk of getting cancer” and “I feel guilty that I might pass on LFS to my children.” Adapted items were reviewed for clarity by the research team and the original author. Responses were measured on a 5-point Likert scale (1 = “strongly disagree,” 3 = “neither agree nor disagree,” 5 = “strongly agree”). A 10-item composite measure was calculated, with sum scores ranging from 10 to 50 and higher scores indicating a greater level of concern about the consequences of LFS (Supplementary Table S1). Excluding the two child-related items (items 9 and 10, Table S1), corrected item–total correlations ranged from 0.44 to 0.77. As expected, the two child-related items showed weaker correlations (*r* = 0.02–0.04) but were retained due to their conceptual relevance. Internal consistency for the full 10-item scale was α = 0.79.

**Health-related quality of life**

We measured HRQOL over the past month using a slightly modified version of the World Health Organization WHOQOL-BREF measure [32], as described in our previous work [7]. Three separate HRQOL domains were assessed: general satisfaction with HRQOL (2 items: e.g., “I am satisfied with my health”), termed general HRQOL hereafter, mental HRQOL (5 items: e.g., “My life is meaningful”), and physical HRQOL (7 items: e.g., “When I need to, I am able to get around”). Responses were measured on a 5-point Likert scale (1 = “strongly disagree,” 3 = “neither agree nor disagree,” 5 = “strongly agree”). Responses were averaged to create three composite domain scores. Mean domain scores were multiplied by four to enable comparison to WHOQOL-100 scores (range = 0–100), the long-form WHO assessment of HRQOL. For general HRQOL, the inter-item correlation was *r* = 0.66, *p* < 0.001, and internal consistency was α = 0.79. Corrected item-total correlations ranged from 0.43 to 0.86 for physical HRQOL and 0.50 to 0.73 for mental HRQOL, with internal consistency α = 0.86 for both domains.

##### History of mental health challenges

History of mental health challenges was measured using three items from the GPRI [29], chosen to minimize respondent burden and potential distress associated with more comprehensive mental health measures in this vulnerable population. The GPRI was developed and validated to assess psychosocial factors contributing to distress in the context of hereditary disease, with mental health history identified as a key construct in its three-factor structure. Example items include “I have been diagnosed with a depressive or anxiety disorder in the past” and “I have had emotional problems in the past that led me to have thoughts about suicide” (*yes*/*no*). A 3-item composite measure was calculated, with sum scores ranging from 0 to 3 and higher scores indicating greater mental health challenges. The composite measure had an average inter-item correlation of *r* = 0.29 (range = 0.24–0.32) and an internal consistency of α = 0.78.

### Clinical and demographic data

We collected data on several clinical-demographic characteristics (Table 1). These included sex, age, age at genetic testing for LFS, prior cancer history, age at first primary cancer, time between first primary cancer and the survey, has children, number of first-degree relatives (FDRs) with cancer, bereavement of FDRs, and age at bereavement of FDRs. Bereavement of FDRs was measured with the item, “I lost a close family member (e.g., parent/sibling) to the disease for which I am receiving counseling/testing” (*yes*/*no*). Personal and family cancer history and genetic testing information were extracted from NCI LFS study records. Additional clinical-demographic information for this survey sample (race/ethnicity, educational attainment) has been reported elsewhere [7].

**Table 1:** Participant clinical-demographics.

Variable (*n* = 37)	
Age	
Mean (SD)	31 (6.0)
Range	19–39
Age at genetic testing for LFS^a^	
Mean (SD)	24 (6.4)
Range	12–35
Sex (*n*, %)	
Male	6 (16)
Female	31 (84)
Has children (*n*, %)	
No	24 (65)
Yes	13 (35)
Cancer status	
No cancer (*n*, %)	18 (49)
One or more primary cancers (*n*, %)	19 (51)
Age at first primary cancer diagnosis (*n* = 19)	
Mean (SD)	23 (9.4)
Range	0–35
Family cancer history	
Number of FDRs with cancer history	
Mean (SD)	1.41 (0.86)
Range	0–3
FDR bereavement (*n*, %)	19 (51)
Age at FDR bereavement (*n* = 19)	
Mean (SD)	15.5 (10.1)
Range	2–37
Mental health history (*n*, % agree with each statement)	
I have had emotional problems in the past.	26 (70)
I have been diagnosed with a depressive or anxiety disorder in the past.	25 (68)
I have had emotional problems that led me to have thoughts about suicide.	16 (43)

*FDR* First-degree relative. All age and time-related variables are reported in years.

^a^Missing data: *n* = 1. Median value for this item imputed for this participant.

### Statistical analysis

We used RStudio (version 2023.6.1.524) to calculate descriptive statistics and assess relationships between variables using correlational and regression analyses. Correlational analyses were conducted to examine associations between psychosocial and clinical-demographic variables and guide variable selection for regression models. Due to specific interest in investigating mental health among YAs, we also disaggregated items from the history of mental health challenges variable to explore relationships among each of these items, CC-LFS, and clinical-demographic factors. In addition, we used simple linear regression analysis to assess associations between CC-LFS and each HRQOL domain and CC-LFS and the history of mental health challenges sum score. Hierarchical multiple linear regression analysis was then performed to examine associations between CC-LFS and physical, mental, and general HRQOL while controlling for variables known to affect HRQOL based on correlation analyses and supporting literature (i.e., age, prior cancer history, and history of mental health challenges) [20, 29, 33, 34]. Three separate models were constructed, each using a different HRQOL domain as the dependent variable and CC-LFS as the independent variable. Variables were added stepwise in the following order: (1) clinical-demographic factors (age, prior cancer history), (2) history of mental health challenges, and (3) CC-LFS. Change in adjusted *R*^*2*^ was assessed at each step to determine how much variability in HRQOL could be explained by the added variable(s). Due to sample size constraints, we included all participants in regression models controlling for rather than stratifying by covariates. We calculated standardized coefficients (β) with 95% confidence intervals (CI). Statistical significance was set at *p* < 0.05.

## Results

### Study participants

In total, 37 out of 112 invited participants completed the survey (*M*_age_ = 31 years, SD = 6.0), for a response rate of 33%. Participants were predominantly female (*n* = 30, 81%, reflecting invited participants who were 71% female) and about half reported a prior cancer history (*n* = 19, 51%). Average age at genetic testing was 24 years (SD = 6.4, range = 12–35), with mean time between genetic testing and survey participation 6.9 years (SD = 3.7, range = 1–16). Additional clinical-demographic characteristics are displayed in Table 1.

### Psychosocial variables

Descriptive statistics are reported in Table 2. All psychosocial variables were approximately normally distributed, except for the HRQOL domains, which were negatively skewed (Table 2). The CC-LFS demonstrated adequate variability (*M* = 31 out of 50, SD = 7.2). Floor and ceiling effects were minimal, with only 2.7% of responses at the lowest and highest possible scores, respectively. Items 1 and 8 focused on concern about cancer risk and implications of developing cancer and were the highest scoring: 81% (*n* = 30) and 89% (*n* = 33) of respondents agreed or strongly agreed with each item, respectively. Items 4 and 5 assessed whether cancer risk impacted close relationships and were the lowest scoring: 73% (*n* = 27) and 57% (*n* = 21) of respondents disagreed or strongly disagreed with each item, respectively (Supplementary Table S1). Mean scores were as follows for general HRQOL (*M* = 63.9, SD = 26.0), mental HRQOL (*M* = 65.8, SD = 18.2), and physical HRQOL (*M* = 67.5, SD = 21.7). Mean mental and physical HRQOL scores were lower than reported estimates in the general population [35, 36]. The mean history of mental health challenges sum score was 1.8 out of 3 (SD = 1.2). Most participants reported past emotional problems (*n* = 26/37, 70%) or diagnoses of depressive and/or anxiety disorders (*n* = 25/37, 68%), and nearly half reported past emotional problems that led to thoughts about suicide (*n* = 16/37, 43%) (Table 1).

**Table 2:** Descriptive statistics of study variables.

Psychosocial variables	Mean	Median	SD	Range^b^
Concerns about the consequences of LFS sum score (α = 0.79)	30.9	31	7.2	17–46
1. If I learn that I have cancer, I believe that I will have more problems in my life.	4.1	4	0.9	1–5
2. If I learn that I have cancer, I believe that I will change plans for my career/profession.	3.3	4	1.1	1–5
3. If I learn that I have cancer, I believe that I will have difficulties in my family relationships.	2.5	3	1.4	1–5
4. My cancer risk is currently causing a significant disruption in my family life.^a^	2.2	2	1.3	1–5
5. I am worried that my cancer risk will impact my relationship with my significant other (or future partner).	2.7	2	1.5	1–5
6. My worries about cancer affect my daily mood.	2.6	3	1.3	1–5
7. I worry often about my risk of getting cancer.	3.4	4	1.2	1–5
8. I am concerned about my risk of getting cancer.	4.1	4	0.9	1–5
9. I am worried about talking to my children (young or adult) about the heritable nature of LFS.	2.8	3	1.1	1–5
10. I feel guilty that I might pass on LFS to my children.	3.2	3	1.3	1–5
Health-related quality of life				
General HRQOL (α = 0.79)	63.9	75	26	0–100
Physical HRQOL (α = 0.86)	67.5	69	21.7	6–100
Mental HRQOL (α = 0.86)	65.8	69	18.3	6–94
Mental health history (α = 0.78)	1.8	2	1.2	0–3

^a^Missing data: *n* = 1. Median value for this item imputed for this participant.

^b^Response scales per measure: CC-LFS sum score, 10–50; single CC-LFS items, 1–5; HRQOL domains, 0–100 after transformation; Mental health history, 0–3: sum score of 3x yes (1) or no (0) questions.

### Relationships among psychosocial and clinical-demographic variables

In correlational analysis of psychosocial and clinical-demographic factors (Supplementary Table S2), CC-LFS was negatively associated with general HRQOL (*r* = −0.54, 95% CI −0.73, −0.26, *p* < 0.001), mental HRQOL (*r* = −0.36, 95% CI −0.61, −0.04, *p* = 0.031), and physical HRQOL (*r* = −0.59, 95% CI −0.77, −0.33, *p* < 0.001). CC-LFS was positively associated with history of mental health challenges (*r* = 0.34, 95% CI 0.02, 0.60, *p* = 0.037). There were negative associations between history of mental health challenges and each HRQOL domain and history of suicidal ideation and each HRQOL domain (Supplementary Table S2). Age and mental HRQOL were positively associated (*r* = 0.35, 95% CI 0.03, 0.61, *p* = 0.031), such that older participants reported better mental HRQOL. Clinical-demographic and psychosocial factors were otherwise not significantly correlated (Supplementary Table S2).

### Regression analyses: associations with HRQOL

Simple regression analysis indicated that CC-LFS was negatively associated with each HRQOL domain: general HRQOL (β = −0.536, 95% CI −0.83, −0.25, *R*^2^_adj_ = 0.266, F(1,35) = 14.08, *p* < 0.001), mental HRQOL (β = −0.355, 95% CI −0.68, −0.03, *R*^2^_adj_ = 0.101, F(1,35) = 5.056, *p* = 0.031), and physical HRQOL (β = −0.594, 95% CI −0.87, −0.32, *R*^2^_adj_ = 0.334, F(1,35) = 19.07, *p* < 0.001). History of mental health challenges was also negatively associated with general HRQOL (β = −0.427, 95% CI −0.74, −0.12, *R*^2^_adj_ = 0.159, F(1,35) = 7.784, *p* = 0.008), mental HRQOL (β = −0.480, 95% CI −0.78, −0.18, *R*^2^_adj_ = 0.208, F(1,35) = 19.07, *p* = 0.003), and physical HRQOL (β = −0.594, 95% CI −0.87, −0.32, *R*^2^_adj_ = 0.334, F(1,35) = 5.007, *p* < 0.001).

Multiple regression analysis results are reported in Table 3. In the final models, controlling for prior cancer history, age, and history of mental health challenges, higher CC-LFS scores were associated with poorer general HRQOL (β = −0.466, 95% CI −0.77, −0.16, *p* = 0.004). CC-LFS scores (β = −0.491, 95% CI −0.76, −0.22, *p* < .001) and history of mental health challenges (β = −0.385, 95% CI −0.65, −0.12, *p* = 0.006) were negatively associated with physical HRQOL. CC-LFS scores accounted for a statistically significant proportion of unique variance in general HRQOL (Δ*R*^2^_adj_ = 17.7%, *p* = 0.004) and physical HRQOL (Δ*R*^2^_adj_ = 20.4%, *p* < 0.001). The relationship between CC-LFS and mental HRQOL was nonsignificant (*p* = 0.102); however, age (β = 0.318, 95% CI 0.03, 0.61, *p* = 0.031) and history of mental health challenges (β = −0.353, 95% CI −0.66, −0.05, *p* = 0.025) were significantly associated, such that older aged YAs and those with fewer past mental health problems reported better mental HRQOL.

**Table 3:** Hierarchical multilinear regressions modeling associations between concerns about the consequences of LFS and health-related quality of life.

Model 1. Outcome: general health-related quality of life																	
Predictor	Step 1					Step 2						Step 3					
	β	SE	*t*	*p*	*R*^2^	β	SE	*t*	*p*	*R*^2^	Δ*R*^*2*^	β	SE	*t*	*p*	*R*^2^	Δ*R*^*2*^
Cancer history	−0.047	0.335	−0.142	0.888	0.035	−0.090	0.309	−0.291	0.773	0.131	0.096	0.092	0.282	0.325	0.747	0.308^**^	0.177^**^
Age	0.187	0.170	1.10	0.280		0.145	0.157	0.924	0.362			0.168	0.141	1.20	0.240		
Mental health						−0.414	0.156	−2.65	**0.012**			−0.246	0.150	−1.64	0.112		
CC-LFS												−0.466	0.151	−3.07	**0.004**		

Model 2. Outcome: mental health-related quality of life																	
Predictor	Step 1					Step 2						Step 3					
	β	SE	*t*	*p*	*R*^2^	β	SE	*t*	*p*	*R*^2^	Δ*R*^*2*^	β	SE	*t*	*p*	*R*^2^	Δ*R*^*2*^
Cancer history	0.072	0.319	0.228	0.821	0.076	0.026	0.285	0.093	0.927	0.262^**^	0.186	0.127	0.284	0.447	0.658	0.301^**^	0.039
Age	0.350	0.161	2.17	**0.037**		0.305	0.145	2.11	**0.043**			0.318	0.141	2.25	**0.031**		
Mental health						−0.446	0.144	−3.10	**0.004**			−0.353	0.151	−2.34	**0.025**		
CC-LFS												−0.256	0.152	−1.68	0.102		

Model 3. Outcome: physical health-related quality of life																	
Predictor	Step 1					Step 2						Step 3					
	β	SE	*t*	*p*	*R*^2^	β	SE	*t*	*p*	*R*^2^	Δ*R*^*2*^	β	SE	*t*	*p*	*R*^2^	Δ*R*^*2*^
Cancer history	0.178	0.339	0.525	0.603	0.01	0.120	0.285	−0.421	0.676	0.265^**^	0.264	0.312	0.247	1.26	0.217	0.469^***^	0.204^***^
Age	0.062	0.172	0.362	0.720		0.006	0.145	0.042	0.967			0.030	0.123	0.246	0.807		
Mental health						−0.563	0.144	−3.91	**0.000**			−0.385	0.131	−2.93	**0.006**		
CC-LFS												−0.491	0.133	−3.70	**0.000**		

Cancer history (1= cancer). *R*^2^ are adjusted values and refer to model fit at each step. Δ*R*^*2*^ denotes change in fit between steps and if unique variance added is statistically significant.

*CC-LFS* concerns about the consequences of LFS, *Mental health* mental health history sum score.

*P*-values in bold denote *p* ≤ 0.05, otherwise **p ≤ 0.01; ***p ≤ 0.001.

**Fig. 1:**
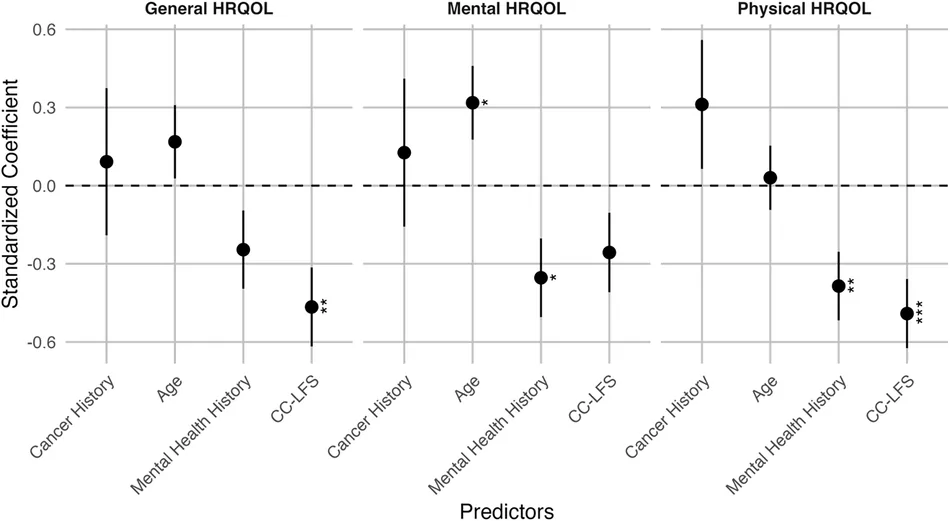
Final multilinear regression models showing associations between concerns about the consequences of LFS and health-related quality of life. CC-LFS concerns about the consquences of LFS; mental health history, sum score. Cancer history (1 = cancer). *p ≤ 0.05; **p ≤ 0.01; ***p ≤ 0.001.

Figure 1 depicts the final models for each HRQOL domain. Final models explained a significant amount of variance in HRQOL, accounting for 30.8% of the variance in general HRQOL (*R*^2^_adj_ = 0.308, F(4,32) = 5.007, *p* = 0.003, power = 0.77), 46.9% of the variance in physical HRQOL (*R*^2^_adj_ = 0.469, F(4,32) = 8.944, *p* < 0.001, power = 0.92), and 30.1% of the variance in mental HRQOL (*R*^2^_adj_ = 0.301, F(4,32) = 4.871, *p* = 0.004, power = 0.76). Due to the observed correlations between study variables (Supplementary Table S2), we tested for and found no significant multicollinearity; variance inflation factors ranged from 1.03 to 1.19 for each final model and were below the established cut-off scores of 5 or 10 [37].

## Discussion

This study assessed YAs’ concerns about the consequences of LFS and how those concerns relate to HRQOL and mental health challenges. Participants reported a range of LFS-related concerns, especially regarding their cancer risk and the potential impact a diagnosis could have on their career/profession or life plans. In line with our hypothesis, greater concern—as measured by CC-LFS— was associated with lower general and physical HRQOL. Notably, CC-LFS scores explained a significant proportion of unique variance in both domains (17.7% and 20.4%, respectively), suggesting that concerns about LFS may exert a meaningful and independent effect on wellbeing. A reverse causal direction, however, cannot be discounted; it is also possible that lower HRQOL intensifies YAs’ concerns about LFS. These findings highlight that concerns about cancer predisposition may be an enduring psychosocial risk factor in the YA LFS population. The average time since genetic testing in this sample was approximately seven years, indicating that these concerns are not limited to the time surrounding disclosure of genetic testing results but may persist or evolve well into survivorship and adulthood.

Although CC-LFS was associated with general and physical HRQOL, it was not significantly associated with mental HRQOL after adjusting for other variables. However, younger age and more past mental health challenges were associated with lower mental HRQOL, suggesting that individuals with these characteristics may be at higher psychosocial risk [20, 29, 34]. We also observed that history of mental health challenges was negatively associated with physical HRQOL, which suggests that YAs with functional limitations, pain, or fatigue due to LFS-related cancers or comorbidities may have heightened psychosocial risk, similar to AYA cancer survivors [38, 39]. Together, these findings underscore the importance of addressing both mental and physical domains of quality of life in this population.

This study advances psychosocial research in young people with LFS by quantifying LFS-related concerns using an adapted version of the GPRI [29]. YAs reported a range of concerns about the consequences of LFS: concerns about developing cancer were the most frequently endorsed whereas concerns about the relational impact of their cancer risk were endorsed least often. This pattern reflects previous qualitative findings highlighting the salience of cancer-specific distress among young people with LFS [8, 12, 13], and aligns with research documenting elevated levels of cancer worry and fear of progression among adults with LFS [24, 26]. Notably, variability in concerns may have important implications for HRQOL. For example, fear of progression and/or recurrence have been associated with reduced HRQOL in adults with LFS [26], and both AYA and adult cancer survivors [40, 41]. Given the psychosocial burden of cancer worries and fear, monitoring YAs’ specific concerns about cancer predisposition is important in long-term management of LFS.

Our finding that CC-LFS, but not prior cancer history, was associated with all domains of HRQOL and mental health challenges suggests that concerns about living with LFS may adversely affect wellbeing even in the absence of a personal cancer diagnosis. This aligns with previous research showing that cancer-related concerns (i.e., fear of progression) are associated with reduced mental and physical HRQOL in adults with LFS, independent of cancer history [26]. Together, these findings highlight the distinct and enduring psychosocial needs of individuals living with LFS, regardless of whether they have personally experienced cancer [18]. Longitudinal research is needed to clarify which concerns most strongly impact HRQOL over time and how these concerns shift across different phases of LFS (e.g., diagnosis, cancer treatment, long-term adaptation) [8] or over the life course, as successive developmental stages pique different concerns (e.g., family formation prompting concerns about heredity) [9].

Our findings reinforce that young adulthood is a vulnerable period for psychosocial health among individuals with LFS. In our sample, half of participants reported early life experiences of cancer and/or the loss of a parent or sibling—both established risk factors for distress and long-term mental health issues [42–44]. Mental health challenges were highly prevalent: 68% of participants reported a diagnosis of anxiety and/or depression, and 43% reported past suicidal ideation. These rates far exceed those reported in a previous GPRI study of adults being evaluated for cancer- and non-cancer-related hereditary genetic conditions (25% reported anxiety and/or depression diagnoses and 14% past suicidal ideation) [30], and also surpass pooled prevalence estimates among AYA cancer survivors from a large meta-analysis (29% for anxiety; 24% for depression) [45]. Although data on suicidal ideation in hereditary cancer syndromes are limited, the rate in our sample mirrors findings from a small AYA survivor cohort (38%) [46]. These results highlight the need to better characterize the onset, course, and clinical features of mental health issues in LFS. Larger, longitudinal studies using standardized instruments (e.g., GAD-7, PHQ-9, or PROMIS) would strengthen measurement consistency and support cross-study comparisons, e.g. [47].

### Clinical implications

Diminished HRQOL appears to be a risk factor for YAs well beyond initial diagnosis and genetic counseling for LFS, reinforcing the need for accessible, long-term psychosocial support [8, 10, 12, 15, 19, 23]. Lower mental HRQOL was associated with younger age, therefore, identifying and addressing psychosocial risk factors among young people with LFS early and with developmentally appropriate psychosocial care is crucial to protecting mental HRQOL. Past mental health challenges were prevalent in our sample and were a notable correlate of both reduced mental and physical HRQOL, supporting calls to integrate mental health screening and support in hereditary cancer settings [47]. Our findings also suggest that suicide-related distress in this population may be clinically significant. Ideation is an important precursor to suicidal intent, attempts, and deaths and represents a critical target for clinical attention as suicide death rates have increased among young cancer survivors [48]. Though traditionally used at the time of genetic testing, psychosocial screening tools for use in genetics (e.g., GPRI), with adaptations as needed, show promise for use across the continuum of care. These tools may help identify individuals at risk of distress and guide tailored support in multidisciplinary cancer risk management and long-term follow-up settings.

### Strengths and limitations

This study was strengthened by its focus on an underserved population of young people with distinctive unmet psychosocial needs due to inherited multi-organ cancer risk. Our sample size was limited given the rarity of LFS and our age-limited criteria. Nonetheless, post-hoc power analyses (*n* = 37, α = 0.05) demonstrated we had moderate to sufficient power to detect observed effects, many of which were highly statistically significant with meaningful effect sizes. Although the measures were based on validated instruments, slight modifications were made for contextual relevance and have not been formally re-validated, which may affect direct comparability with prior research. The cross-sectional design limits causal inference and prospective studies with larger samples are needed to improve generalizability and test indirect and interaction effects. Our findings may have been influenced by having a mostly female sample (reflecting the NCI LFS study YAs at the time of the study), who are at higher risk of ongoing mental health challenges into young adulthood [49] and LFS-related cancers at earlier ages [1]. This study also took place during the COVID-19 pandemic; a challenging period for young people globally in terms of mental health [50].

## Conclusion

This study improves our understanding of concerns about the consequences of LFS and how they relate to HRQOL and mental health in YAs with LFS. YAs’ concerns about LFS may affect general and physical aspects of their HRQOL, while mental health challenges further impact both mental and physical HRQOL. Our findings align with existing evidence on individuals’ psychosocial concerns of LFS, highlighting their potential negative impact on well-being during young adulthood. Given the high, lifelong cancer risks associated with LFS, and potential for reduced HRQOL beyond initial diagnosis, ongoing psychosocial support is essential. Our study identifies areas for future research, particularly in developing long-term supportive care interventions tailored to YAs’ concerns about LFS. Effectively managing the psychosocial challenges of LFS requires interdisciplinary care teams, including mental health providers, genetic counselors, and physicians.

## Supplementary information

Supplementary Table 1

Supplementary Table 2

Supplementary Material 3

## Data Availability

De-identified data will be shared with qualified investigators after IRB review and establishment of appropriate data transfer agreements. Please contact the corresponding author for data requests.

## References

[1] de Andrade KC, Khincha PP, Hatton JN, Frone MN, Wegman-Ostrosky T, Mai PL, et al. Cancer incidence, patterns, and genotype-phenotype associations in individuals with pathogenic or likely pathogenic germline TP53 variants: an observational cohort study. Lancet Oncol. 2021;22:1787–98. 34780712 10.1016/S1470-2045(21)00580-5 PMC8915249

[2] Mai PL, Best AF, Peters JA, DeCastro RM, Khincha PP, Loud JT, et al. Risks of first and subsequent cancers among TP53 mutation carriers in the National Cancer Institute Li-Fraumeni syndrome cohort. Cancer. 2016;122:3673–81. 27496084 10.1002/cncr.30248 PMC5115949

[3] Kratz CP, Achatz MI, Brugières L, Frebourg T, Garber JE, Greer M-LC, et al. Cancer Screening recommendations for individuals with Li-Fraumeni syndrome. Clin Cancer Res. 2017;23:e38–45. 28572266 10.1158/1078-0432.CCR-17-0408

[4] National Comprehensive Cancer Network. Genetic/Familial High-Risk Assessment: Breast, Ovarian, Pancreatic, and Prostate: NCCN Clinical Practice Guidelines in Oncology; Version 2. 2025. Updated November 2024. Plymouth Meeting, PA: National Comprehensive Cancer Network; 2024. 10.6004/jnccn.2021.0001 33406487

[5] Forbes Shepherd R, Rising CJ, Huelsnitz CO, Boyd P, Wilsnack C, Sleight AG, et al. Mental health of adolescents and young adults withLi-Fraumeni syndrome: results from a mixed method study (P24.013.C). [Conference Abstract from the 55th European Society of Human Genetics (ESHG) Conference: Hybrid Posters]. Eur J Hum Genet. 2023;31:345–709.36564538

[6] Shepherd RF, Rising CJ, Boyd P, Wilsnack C, Sleight AG, Thompson AS, et al. Mental health outcomes for young people with inherited cancer syndromes: a mixed-method study of Li-Fraumeni syndrome. Ann Behav Med. 2023;57:S265–S.

[7] Rising CJ, Huelsnitz CO, Shepherd RF, Klein WMP, Sleight AG, Wilsnack C, et al. Diet and physical activity behaviors: how are they related to illness perceptions, coping, and health-related quality of life in young people with hereditary cancer syndromes? J Behav Med. 2024;47:707–20. 38642305 10.1007/s10865-024-00489-z PMC11291531

[8] Forbes Shepherd R, Werner-Lin A, Keogh LA, Delatycki MB, Forrest LE. “I need to know if I’m going to die young”: Adolescent and young adult experiences of genetic testing for Li–Fraumeni syndrome. J Psychosoc Oncol. 2021;39:54–73. 32449501 10.1080/07347332.2020.1768199

[9] Forbes Shepherd R, Werner-Lin A, Keogh LA, Delatycki MB, Forrest LE. Reproduction and genetic responsibility: an interpretive description of reproductive decision-making for young people with Li-Fraumeni syndrome. Qualitative Health Res. 2022;32:168–81. 10.1177/10497323211046240 34781775

[10] Rising CJ, Wilsnack C, Boyd P, Sleight AG, Hutson SP, Khincha PP, et al. Family communication challenges of adolescents and young adults with Li-Fraumeni syndrome: Implications for psychosocial care. Patient Educ Counsel. 2022;105:3259–66. 10.1016/j.pec.2022.07.012 PMC9529832 35918231

[11] Sleight AG, Rising CJ, Boyd P, Wilsnack C, Goodfellow M, Khincha PP, et al. “I can control what I do with my daily life”: Occupational experiences of adolescents and young adults with Li-Fraumeni Syndrome. J Occup Sci. 2022;30:661–72.

[12] Forbes Shepherd R. Coming of age with Li-Fraumeni syndrome: perspectives of young people and health professionals. Melbourne, Australia: The University of Melbourne; 2020.

[13] Forbes Shepherd R, Keogh L, Werner-Lin A, Delatycki MB, Forrest LE. Benefits and burdens of risk management for young people with inherited cancer: a focus on Li-Fraumeni syndrome. Aust J Gen Pr. 2021;50:538–44. 10.31128/AJGP-04-21-5954 34333565

[14] Werner-Lin A, Forbes Shepherd R, Young JL, Wilsnack C, Merrill SL, Greene MH, et al. Embodied risk for families with Li-Fraumeni syndrome: Like electricity through my body. Soc Sci Med. 2022;301:114905. 35367908 10.1016/j.socscimed.2022.114905 PMC9237847

[15] Rising CJ, Forbes Shepherd R, Sleight AG, Boyd P, Wilsnack C, Thompson AS, et al. Relating to the body under chronic cancer threat: implications for psychosocial health among adolescents and young adults with cancer predisposition syndromes. J Adolesc Young Adult Oncol. 2025;14:151–9. 39331584 10.1089/jayao.2024.0103 PMC12171651

[16] Ross J, Bojadzieva J, Peterson S, Noblin SJ, Yzquierdo R, Askins M, et al. The psychosocial effects of the Li-Fraumeni Education and Early Detection (LEAD) program on individuals with Li-Fraumeni syndrome. Genet Med. 2017;19:1064–71. 28301458 10.1038/gim.2017.8 PMC5875687

[17] Forbes Shepherd R, Huelsnitz CO, Khincha PP, Han PKJ. Understanding scanxiety among individuals with Li-Fraumeni syndrome undergoing periodic cancer screening: a qualitative study (PA6-4). [Conference Abstract from The 17th International Meeting on Psychosocial Aspects of Hereditary Cancer (IMPAHC)]. Fam Cancer. 2024;23:694.

[18] Werner-Lin A, Forbes Shepherd R, Rising CJ, Thompson AS, Huelsnitz C, Wilsnack C, et al. How do young people with a hereditary cancer predisposition syndrome understand and experience cancer survivorship? “With Li-Fraumeni syndrome, it’s just an intermission”. Psycho Oncol. 2023;32:375–82. 10.1002/pon.6080 36514197

[19] Werner-Lin A, Young JL, Wilsnack C, Merrill SL, Groner V, Greene MH, et al. Waiting and “weighted down”: the challenge of anticipatory loss for individuals and families with Li-Fraumeni Syndrome. Fam Cancer. 2020;19:259–68. 32222840 10.1007/s10689-020-00173-6 PMC7440840

[20] Gopie JP, Vasen HFA, Tibben A. Surveillance for hereditary cancer: Does the benefit outweigh the psychological burden?—A systematic review. Crit Rev Oncol Hematol. 2012;83:329–40. 22366115 10.1016/j.critrevonc.2012.01.004

[21] Brand H, Speiser D, Besch L, Roseman J, Kendel F. Making sense of a health threat: illness representations, coping, and psychological distress among BRCA1/2 mutation carriers. Genes. 2021;12:741. 34069035 10.3390/genes12050741 PMC8156260

[22] van Lier MG, Mathus-Vliegen EM, van Leerdam ME, Kuipers EJ, Looman CW, Wagner A, et al. Quality of life and psychological distress in patients with Peutz-Jeghers syndrome. Clin Genet. 2010;78:219–26. 20695872 10.1111/j.1399-0004.2010.01469.x

[23] Forbes Shepherd R, Lewis A, Keogh LA, Werner-Lin A, Delatycki MB, Forrest LE. A systematic review of how young people live with inherited disease: what can we learn for Li-Fraumeni syndrome? J Adolesc Young Adult Oncol. 2018;7:525–45. 30004834 10.1089/jayao.2018.0028

[24] Lammens CR, Aaronson NK, Wagner A, Sijmons RH, Ausems MG, Vriends AH, et al. Genetic testing in Li-Fraumeni syndrome: uptake and psychosocial consequences. J Clin Oncol. 2010;28:3008–14. 20479422 10.1200/JCO.2009.27.2112

[25] Peters JA, Kenen R, Bremer R, Givens S, Savage SA, Mai PL. Easing the burden: describing the role of social, emotional and spiritual support in research families with Li-Fraumeni syndrome. J Genet Counsel. 2016;25:529–42. 10.1007/s10897-015-9905-x 26621765

[26] Kiermeier S, Schott S, Nees J, Dutzmann C, Struwe F, Kratz CP, et al. Health-related quality of life and fear of progression in individuals with Li-Fraumeni syndrome. J Genet Counsel. 2025;34:e1859. 10.1002/jgc4.1859 PMC11726407 38348940

[27] Cella D, Hughes C, Peterman A, Chang CH, Peshkin BN, Schwartz MD, et al. A brief assessment of concerns associated with genetic testing for cancer: the Multidimensional Impact of Cancer Risk Assessment (MICRA) questionnaire. Health Psychol. 2002;21:564–72. 12433008

[28] Eijzenga W, Bleiker EMA, Hahn DEE, Kluijt I, Sidharta GN, Gundy C, et al. Psychosocial Aspects of Hereditary Cancer (PAHC) questionnaire: development and testing of a screening questionnaire for use in clinical cancer genetics. Psycho Oncol. 2014;23:862–9. 10.1002/pon.3485 24443031

[29] Esplen MJ, Cappelli M, Wong J, Bottorff JL, Hunter J, Carroll J, et al. Development and validation of a brief screening instrument for psychosocial risk associated with genetic testing: a pan-Canadian cohort study. BMJ Open. 2013;3:e002227. 10.1136/bmjopen-2012-002227 PMC3612753 23485718

[30] Monohan K, Purvis R, Sexton A, Kentwell M, Thet M, Stafford L, et al. Assessing the acceptability, feasibility, and usefulness of a psychosocial screening tool to patients and clinicians in a clinical genetics service in Australia. J Genet Counsel. 2022;31:653–62. 10.1002/jgc4.1532 34788484

[31] Gomes P, Ferreira T, Mena Matos P, Silva E, Silva J, Esplen MJ, et al. The Genetic Psychosocial Risk Instrument (GPRI): A Validation Study for European Portuguese. Acta Méd Portuguesa. 2022;36:153–61. 10.20344/amp.16497 35793339

[32] World Health Organization. Programme on mental health: WHOQOL user manual, 2012 revision. Geneva: World Health Organization; 1998. Contract No.: WHO/HIS/HSI Rev.2012.03.

[33] LeMasters T, Madhavan S, Sambamoorthi U, Kurian S. A population-based study comparing HRQoL among breast, prostate, and colorectal cancer survivors to propensity score matched controls, by cancer type, and gender. Psycho Oncol. 2013;22:2270–82. 10.1002/pon.3288 PMC4892175 23606210

[34] Meiser B. Psychological impact of genetic testing for cancer susceptibility: an update of the literature. Psycho Oncol. 2005;14:1060–74. 10.1002/pon.933 15937976

[35] Hawthorne G, Herrman H, Murphy B. Interpreting the WHOQOL-BREF: preliminary population norms and effect sizes. Soc Indic Res. 2006;77:37–59.

[36] Noerholm V, Groenvold M, Watt T, Bjorner JB, Rasmussen NA, Bech P. Quality of life in the Danish general population – normative data and validity of WHOQOL-BREF using Rasch and item response theory models. Qual Life Res. 2004;13:531–40. 15085925 10.1023/B:QURE.0000018485.05372.d6

[37] Belsley DA, Kuh E, Welsch RE. Regression diagnostics: Identifying influential data and sources of collinearity. Hoboken, NJ: John Wiley & Sons; 2005.

[38] Smith AW, Bellizzi KM, Keegan THM, Zebrack B, Chen VW, Neale AV, et al. Health-related quality of life of adolescent and young adult patients with cancer in the United States: the adolescent and young adult health outcomes and patient experience study. J Clin Oncol. 2013;31:2136–45. 23650427 10.1200/JCO.2012.47.3173 PMC3731979

[39] Zhang A, Urban-Wojcik E, Seewald M, Zebrack B. Mental health trajectories among US survivors of adolescent and young adult cancer as they age. JAMA Netw Open. 2025;8:e2511430. 40388164 10.1001/jamanetworkopen.2025.11430 PMC12090028

[40] Schulte FSM, Chalifour K, Eaton G, Garland SN. Quality of life among survivors of adolescent and young adult cancer in Canada: a young adults with cancer in their prime (YACPRIME) study. Cancer. 2021;127:1325–33. 33332603 10.1002/cncr.33372

[41] Yang Y, Li W, Wen Y, Wang H, Sun H, Liang W, et al. Fear of cancer recurrence in adolescent and young adult cancer survivors: A systematic review of the literature. Psychooncology. 2019;28:675–86. 30703261 10.1002/pon.5013

[42] Lee A, Low CE, Yau CE, Li J, Ho R, Ho CSH. Lifetime burden of psychological symptoms, disorders, and suicide due to cancer in childhood, adolescent, and young adult years: a systematic review and meta-analysis. JAMA Pediatr. 2023;177:790–9. 37345504 10.1001/jamapediatrics.2023.2168 PMC10288378

[43] van Dooren S, Seynaeve C, Rijnsburger AJ, Duivenvoorden HJ, Essink-Bot ML, Bartels CC, et al. The impact of having relatives affected with breast cancer on psychological distress in women at increased risk for hereditary breast cancer. Breast Cancer Res Treat. 2005;89:75–80. 15666200 10.1007/s10549-004-2623-y

[44] Van Oostrom I, Meijers-Heijboer H, Duivenvoorden HJ, Bröcker-Vriends AHJT, Van Asperen CJ, Sijmons RH, et al. Experience of parental cancer in childhood is a risk factor for psychological distress during genetic cancer susceptibility testing. Ann Oncol. 2006;17:1090–5. 16600981 10.1093/annonc/mdl069

[45] Osmani V, Hörner L, Klug SJ, Tanaka LF. Prevalence and risk of psychological distress, anxiety and depression in adolescent and young adult (AYA) cancer survivors: A systematic review and meta-analysis. Cancer Med. 2023;12:18354–67. 37559504 10.1002/cam4.6435 PMC10523984

[46] Giberson SA Jr., Hall BC, Jester B, Short VM, Roaten K, de la Garza N, et al. Suicidal ideation and depression among adolescent and young adult cancer patients. J Adolesc Young Adult Oncol. 2021;10:549–54. 33857381 10.1089/jayao.2020.0180

[47] Kastner AM, Fischer-Jacobs J, Brederecke J, Hahne A, Zimmermann T. Distress, anxiety, and depression in persons with hereditary cancer syndromes: results from a nationwide cross-sectional study in Germany. Cancer Med. 2023;12:13701–11. 37132195 10.1002/cam4.5999 PMC10315723

[48] Matsuo K, Duval CJ, Nanton BA, Yao JA, Yu E, Pino C, et al. Suicide deaths among adolescent and young adult patients with cancer. JAMA Netw Open. 2024;7:e2442964. 39495514 10.1001/jamanetworkopen.2024.42964 PMC11536222

[49] Patton GC, Coffey C, Romaniuk H, Mackinnon A, Carlin JB, Degenhardt L, et al. The prognosis of common mental disorders in adolescents: a 14-year prospective cohort study. Lancet. 2014;383:1404–11. 24439298 10.1016/S0140-6736(13)62116-9

[50] McGorry PD, Mei C, Dalal N, Alvarez-Jimenez M, Blakemore S-J, Browne V, et al. The Lancet Psychiatry Commission on youth mental health. Lancet Psychiatry. 2024;11:731–74. 39147461 10.1016/S2215-0366(24)00163-9

