# Supplementary material for: Concerns about the consequences of cancer predisposition and relationships with quality of life in young adults with Li-Fraumeni syndrome
Source: Eur J Hum Genet. 2025 Nov 23;34(9):1401–8. doi: 10.1038/s41431-025-01984-w (PMC13550586; doi:10.1038/s41431-025-01984-w)
Supplement: Supplementary file 1 [file 41431_2025_1984_MOESM1_ESM.docx]

**Supplementary Table S1.** Response frequencies to the concerns about the consequences of LFS (CC-LFS) measure

|  | Strongly disagree  1 | Somewhat disagree  2 | Neither agree nor disagree  3 | Somewhat agree  4 | Strongly agree  5 |
| --- | --- | --- | --- | --- | --- |
|  | *n* (%) | | | | |
| 1. If I learn that I have cancer, I believe that I will have more problems in my life. | 1 (3) | 1 (3) | 5 (14) | 16 (43) | 14 (38) |
| 2. If I learn that I have cancer, I believe that I will change plans for my career or profession. | 3 (8) | 6 (16) | 8 (22) | 16 (43) | 4 (11) |
| 3. If I learn that I have cancer, I believe that I will have difficulties in my family relationships. | 14 (38) | 4 (11) | 9 (24) | 6 (16) | 4 (11) |
| 4. My cancer risk is currently causing a significant disruption in my family life. | 14 (38) | 13 (35) | 4 (11) | 2 (5) | 4 (11) |
| 5. I am worried that my cancer risk will impact my relationship with my significant other (or future partner). | 11 (30) | 10 (27) | 2 (5) | 8 (22) | 6 (16) |
| 6. I am worried about talking to my children (young or adult) about the heritable nature of LFS. | 11 (30) | 7 (19) | 9 (24) | 7 (19) | 3 (8) |
| 7. My worries about cancer affect my daily mood. | 2 (5) | 9 (24) | 5 (14) | 13 (35) | 8 (22) |
| 8. I worry often about my risk of getting cancer. | 2 (5) | 0 (0) | 2 (5) | 22 (59) | 11 (30) |
| 9. I am concerned about my risk of getting cancer. | 8 (22) | 1 (3) | 21 (57) | 5 (14) | 2 (5) |
| 10. I feel guilty that I might pass on LFS to my children. | 6 (16) | 1 (3) | 16 (43) | 7 (19) | 7 (19) |

Adapted from the Genetic Psychosocial Risk Instrument (Esplen et al., 2013)

Esplen, M. J., et al. (2013). Development and validation of a brief screening instrument for psychosocial risk associated with genetic testing: a pan-Canadian cohort study. *BMJ Open*, *3*(3). https://doi.org/10.1136/bmjopen-2012-002227
