# Supplementary material for: Concerns about the consequences of cancer predisposition and relationships with quality of life in young adults with Li-Fraumeni syndrome
Source: Eur J Hum Genet. 2025 Nov 23;34(9):1401–8. doi: 10.1038/s41431-025-01984-w (PMC13550586; doi:10.1038/s41431-025-01984-w)
Supplement: Supplementary file 2 [file 41431_2025_1984_MOESM2_ESM.docx]

**Supplementary Table S2.** Bivariate correlations among study variables

|  | 1 | 2 | 3 | 4 | 5 | 5a | 5b | 5c | 6 | 7 | 8 | 9 | 10 |
| --- | --- | --- | --- | --- | --- | --- | --- | --- | --- | --- | --- | --- | --- |
| **Psychosocial variables** |  |  |  |  |  |  |  |  |  |  |  |  |  |
| 1. CC-LFS | - |  |  |  |  |  |  |  |  |  |  |  |  |
| 2. General HRQOL | -.54^**^ | - |  |  |  |  |  |  |  |  |  |  |  |
| 3. Mental HRQOL | -.36^*^ | .77^**^ | - |  |  |  |  |  |  |  |  |  |  |
| 4. Physical HRQOL | -.59^**^ | .80^**^ | .76^**^ | - |  |  |  |  |  |  |  |  |  |
| 5. Mental health history | .34^*^ | -.43^**^ | -.48^**^ | -.57^**^ | - |  |  |  |  |  |  |  |  |
| a. Emotional problems ^a^ | .16 | -.28 | -.38^*^ | -.49^**^ | .85^**^ | - |  |  |  |  |  |  |  |
| b. Anxiety/depression ^a^ | .36^*^ | -.22 | -.36^*^ | -.39^*^ | .82^**^ | .56^**^ | - |  |  |  |  |  |  |
| c. Suicidal ideation ^a^ | .33^*^ | -.55^**^ | -.46^**^ | -.53^**^ | .83^**^ | .57^**^ | .49^**^ | - |  |  |  |  |  |
| **Clinical-Demographics** |  |  |  |  |  |  |  |  |  |  |  |  |  |
| 6. Prior cancer history ^a^ | .18 | -.00 | .08 | .10 | -.06 | -.04 | .02 | -.13 | - |  |  |  |  |
| 7. Sex ^a^ | .00 | .03 | .26 | .09 | .10 | .14 | .11 | .00 | .36^*^ | - |  |  |  |
| 8. Age | .04 | .18 | .35^*^ | .07 | -.11 | -.02 | .08 | -.31 | .12 | .20 | - |  |  |
| 9. Age at genetic testing for LFS | -.01 | .16 | .32 | .07 | -.10 | .03 | .05 | -.32 | .20 | .31 | .84^**^ | - |  |
| 10. Number of FDRs with cancer | .05 | -.01 | .02 | -.08 | .16 | .24 | -.01 | .16 | -.30 | -.09 | .05 | -.09 | - |
| 11. FDR bereavement ^a^ | .19 | .00 | .06 | -.08 | -.04 | .01 | -.17 | .07 | -.19 | -.25 | .19 | .02 | .58^**^ |

Abbreviations: CC-LFS, Concerns about the perceived consequences of LFS; HRQOL, Health-related quality of life; FDR, First-degree relative.

*p < .05; **p < .01; full p-values and 95% CIs are provided in text.

^a^ Point biserial correlations were calculated.
