# Supplementary material for: Concerns about the consequences of cancer predisposition and relationships with quality of life in young adults with Li-Fraumeni syndrome
Source: Eur J Hum Genet. 2025 Nov 23;34(9):1401–8. doi: 10.1038/s41431-025-01984-w (PMC13550586; doi:10.1038/s41431-025-01984-w)
Supplement: Supplementary file 3 [file 41431_2025_1984_MOESM3_ESM.docx]

**Supplementary Material S3.** Study survey measures

**Concerns about the consequences of Li-Fraumeni syndrome (CC-LFS) survey**

The following questions are about your life experiences and feelings. Please select the option that best describes you.

|  | Strongly disagree  1 | Disagree 2 | Neither agree nor disagree 3 | Agree 4 | Strongly  agree 5 |
| --- | --- | --- | --- | --- | --- |
| 1. If I learn that I have cancer, I believe that I will have more problems in my life. |  |  |  |  |  |
| 2. If I learn that I have cancer, I believe that I will change plans for my career or profession. |  |  |  |  |  |
| 3. If I learn that I have cancer, I believe that I will have difficulties in my family relationships. |  |  |  |  |  |
| 4. My cancer risk is currently causing a significant disruption in my family life. |  |  |  |  |  |
| 5. I am worried that my cancer risk will impact my relationship with my significant other (or future partner). |  |  |  |  |  |
| 6. I am worried about talking to my children (young or adult) about the heritable nature of LFS. |  |  |  |  |  |
| 7. My worries about cancer affect my daily mood. |  |  |  |  |  |
| 8. I worry often about my risk of getting cancer. |  |  |  |  |  |
| 9. I am concerned about my risk of getting cancer. |  |  |  |  |  |
| 10. I feel guilty that I might pass on LFS to my children. |  |  |  |  |  |

Adapted from the Genetic Psychosocial Risk Instrument (Esplen et al., 2013)

**Mental health history survey**

The following questions are about your life experiences and feelings. Please select the option that best describes you.

|  | Yes | No |
| --- | --- | --- |
| 1. I have had emotional problems in the past. |  |  |
| 2. I have been diagnosed with a depressive or anxiety disorder in the past. |  |  |
| 3. I have had emotional problems that led me to have thoughts about suicide. |  |  |

Sourced from the Genetic Psychosocial Risk Instrument (Esplen et al., 2013).

**World Health Organization Quality of Life-Bref items (WHOQOL-Bref) survey**

Over the last month...

| Domain | Item | Strongly disagree  1 | Disagree 2 | Neither agree nor disagree 3 | Agree 4 | Strongly  agree 5 |
| --- | --- | --- | --- | --- | --- | --- |
| G | 1. I am satisfied with my quality of life. |  |  |  |  |  |
| G | 2. I am satisfied with my health. |  |  |  |  |  |
| P | 3. Physical pain prevents me from doing what I need to do. |  |  |  |  |  |
| P | 4. I require medical treatment to function in my daily life. |  |  |  |  |  |
| M | 5. I enjoy life. |  |  |  |  |  |
| M | 6. My life is meaningful. |  |  |  |  |  |
| M | 7. I am able to concentrate. |  |  |  |  |  |
| P | 8. I have enough energy for everyday life. |  |  |  |  |  |
| P | 9. I accept my bodily appearance. |  |  |  |  |  |
| M | 10. When I need to, I am able to get around. |  |  |  |  |  |

During the last month how satisfied have you been...?

| Domain | Item | Very dissatisfied  1 | Dissatisfied  2 | Neither dissatisfied  nor satisfied  3 | Satisfied  4 | Very satisfied  5 |
| --- | --- | --- | --- | --- | --- | --- |
| P | 11. With your sleep? |  |  |  |  |  |
| P | 12. With your ability to perform daily activities? |  |  |  |  |  |
| P | 13. With your capacity to work? |  |  |  |  |  |
| M | 14. With yourself? |  |  |  |  |  |

| Domain | Item | Not at all 1 | Hardly ever  2 | Sometimes  3 | Often  4 | Almost all of the time  5 |
| --- | --- | --- | --- | --- | --- | --- |
| M | 15. How often did you have negative feelings such as blue mood, despair, anxiety, depression? |  |  |  |  |  |

All items adapted from the WHOQOL-Bref (WHO, 1998). G, M, and P denote items from general, mental, and physical HRQOL domains, respectively.

**References**

Esplen, M. J., et al. (2013). Development and validation of a brief screening instrument for psychosocial risk associated with genetic testing: a pan-Canadian cohort study. *BMJ Open*, *3*(3). https://doi.org/10.1136/bmjopen-2012-002227

World Health Organization. Programme on mental health: WHOQOL user manual, 2012 revision. Geneva: World Health Organization; 1998. Contract No.: WHO/HIS/HSI Rev.2012.03.
